# Correlation Between Serum Levels of 25-Hydroxyvitamin D and Severity of Community-Acquired Pneumonia in Hospitalized Patients Assessed by Pneumonia Severity Index: An Observational Descriptive Study

**DOI:** 10.7759/cureus.8947

**Published:** 2020-07-01

**Authors:** Vasiliki E Georgakopoulou, Konstantinos Mantzouranis, Christos Damaskos, Evgenia Karakou, Despoina Melemeni, Dimitrios Mermigkis, Georgios Petsinis, Pagona Sklapani, Nikolaos Trakas, Xanthi Tsiafaki

**Affiliations:** 1 Pulmonology Department, Laiko General Hospital, Athens, GRC; 2 1st Pulmonology Department, Sismanogleio Hospital, Athens, GRC; 3 Renal Transplantation Unit, Laiko General Hospital, Athens, GRC; 4 "N.S. Christeas" Laboratory of Experimental Surgery and Surgical Research, National and Kapodistrian University of Athens Medical School, Athens, GRC; 5 Biochemistry Department, Sismanogleio Hospital, Athens, GRC; 6 Cytopathology Department, Mitera Hospital, Athens, GRC

**Keywords:** community-acquired pneumonia, vitamin d, pneumonia severity index, nutrition, elderly

## Abstract

Introduction

Pneumonia severity index (PSI) is a prognostic index used for estimating the possibility of death due to community-acquired pneumonia. Vitamin D is a fat-soluble vitamin, essential for calcium and phosphate homeostasis. Vitamin D also has antimicrobial properties and according to recent studies, its deficiency may be correlated to an increased frequency of respiratory infections. The serum concentration of 25-hydroxyvitamin D (25(OH)D) is the best vitamin D status index reflecting vitamin D produced in the skin and offered from food and dietary supplements.

Methods

The study involved patients, who fulfilled the criteria of community-acquired pneumonia. The exclusion criteria were: patients <18 years old, severely immunocompromised patients, patients with tuberculosis, patients with malabsorption disorders, nursing home residents, patients with a history of malignancy, chronic renal or liver disease, patients with congestive health failure or cerebrovascular disease, and patients receiving vitamin D as a supplement. The following parameters, recorded on admission, were evaluated: age, sex, co-morbidity, residence in a nursing home, duration of symptoms, clinical symptoms, confusion, blood gas analysis, chest radiograph (pleural effusion), and laboratory parameters. The patients were classified in risk classes according to the PSI. Blood samples were collected within the first 48 hours of hospitalization. The serum levels of 25-hydroxyvitamin D were determined by electrochemiluminescence binding assay in Roche Cobas 601 immunoassay analyzer and mean serum levels of 25-hydroxyvitamin D in each risk class were calculated. For statistical analysis, the statistical program SPSS for Windows version 17.0 (Statistical Package for the Social Sciences, SPSS Inc., Chicago, IL) was used.

Results

A total of 46 patients, 28 males and 18 females, with a mean age of 71.5±17.57 years, hospitalized with community-acquired pneumonia, were included. Sixteen patients (35%) had a severe deficiency, with 25(OH)D levels <10 ng/ml, 17 patients (37%) had moderate deficiency with 25(OH)D levels between 10-20 ng/ml, and 13 patients (28%) had insufficiency with 25(OH)D levels between 20-29 ng/ml. According to the PSI, four (8.7%) patients with a mean age of 53.75±15.43 years were classified as risk class I, 10 (21.7%) patients with a mean age of 54.7±14.82 years as class II, 10 (21.7%) patients with a mean age of 68.41±3.96 years as class III, 17 (37%) patients with a mean age of 84.82±9.73 years as class IV, and five (10.9%) patients with a mean age of 80.2±9.41 years as class V. The mean levels of 25(OH)D were 19.11±11.24 ng/ml in class I, 16.81±8.94 ng/ml in class II, 16.65±9.18 ng/ml in class III, 14.76±10.22 ng/ml in class IV, and 7.49±4.41 ng/ml in class V. There was a positive correlation between low levels of 25(OH)D and the pneumonia severity and statistically significant difference between the mean levels of 25(OH)D in class V (7.49±4.41 ng/ml) compared to overall mean levels in classes I, II, III and IV (16.15±9.49 ng/ml), with p<0.05.

Conclusions

According to our results, there was a positive association between low levels of 25-hydroxyvitamin D and community-acquired pneumonia severity assessed by PSI. The determination of 25-hydroxyvitamin-D status, mostly in patients >60 years old, may prevent severe community-acquired pneumonia.

## Introduction

Vitamin D is a fat-soluble vitamin that is naturally obtained from foods and is offered as a dietary supplement. It is also produced when ultraviolet rays from daylight strike the skin. Vitamin D obtained from sun exposure, food, and dietary supplements is biologically inert and undergoes two hydroxylations for activation. The first hydroxylation occurs in the liver and converts vitamin D to 25-hydroxyvitamin D (25(OH)D), also known as calcidiol, calcifediol, or 25-hydroxyvitamin D3. The second hydroxylation takes place mainly in the kidney and forms the physiologically active 1,25-dihydroxyvitamin D (1,25(OH)2D), additionally called calcitriol [1].

The main role of vitamin D is the enhancement of calcium absorption in the intestine and the maintenance of adequate serum calcium and phosphate levels to enable normal mineralization of bones. It is also useful in bone growth and bone reshaping by osteoblasts and osteoclasts [1-2]. Vitamin D sufficiency helps to prevent rickets in children and osteomalacia in adults and helps to protect older adults from osteoporosis.

Vitamin D has other roles, including modulation of cell growth, neuromuscular and immune function, and reduction of inflammation [1,3-4]. Vitamin D partially modulate numerous genes that encode proteins regulating cell proliferation, differentiation, and apoptosis [1]. The serum concentration of 25(OH)D is the best vitamin D status index, reflecting vitamin D produced in the skin and offered from food and supplements [1], and has a fairly long circulating half-life of 15 days [5].

Vitamin D has long been known to have bactericidal, bacteriostatic, and bacteriolytic properties in vivo and in vitro [6]. Vitamin D response elements are present in promoter regions of the genes encoding for the antimicrobial peptides cathelicidin and beta‐defensin‐2, suggesting that vitamin D plays a role in regulating their expression [7]. Antimicrobial peptides are endogenously synthesized molecules found on the mucosal and epithelial surfaces of all multicellular organisms. They are first-line defense molecules against bacterial and viral infections and they have several other immunomodulatory effects. The two major families of antimicrobial peptides are the defensins, of which there are six alpha and two beta subclasses, and cathelicidins, of which only one subtype exists in humans, the human cathelicidin antimicrobial peptide hCAP18 [8].

Community-acquired pneumonia (CAP) is common worldwide and responsible for significant morbidity and mortality [9]. Risk factors include age, sex, severity of illness, type of pneumonia, comorbidities, and nutritional status [10-11].

A possible protective effect of vitamin D against respiratory tract infections in children and adults has been reported [12-13]. Vitamin D also exerts immunomodulatory effects on these responses to the most common cause of bacterial pneumonia, Streptococcus pneumonia [14], which has been associated with long-term mortality in patients with CAP [15]. The correlation between levels of vitamin D and pneumonia severity assessed by the CURB-65 index, another clinical prediction rule that has been validated for predicting mortality in community-acquired pneumonia, has also been evaluated [16-17].

The pneumonia severity index (PSI) is a clinical prediction rule that medical practitioners can use to calculate the probability of morbidity and mortality among patients with community-acquired pneumonia. The rule uses demographics (whether someone is older and is male or female), the coexistence of co-morbid illnesses, findings on physical examination and vital signs, and essential laboratory findings. According to PSI, patients can be stratified into five risk categories, risk classes I-V, and these classes could be used to predict 30-day survival [18].

Our aim is to evaluate the correlation between serum levels of 25-hydroxyvitamin D and the severity of pneumonia in patients who are hospitalized for community-acquired pneumonia assessed by PSI.

## Materials and methods

The study involved patients who fulfilled the criteria of community-acquired pneumonia. The exclusion criteria were: patients <18 years old, severely immunocompromised patients, patients with tuberculosis, patients with malabsorption disorders, nursing home residents, patients with a history of malignancy, chronic renal or liver disease, patients with congestive health failure or cerebrovascular disease, and patients receiving vitamin D as a supplement, as these factors affect the PSI score and vitamin D levels. The following parameters, recorded on admission, were evaluated: date of presentation (month, year), age, sex, co-morbidity, residence in a nursing home, duration of symptoms, clinical symptoms (body temperature, respiratory rate, heart rate, and arterial systolic and diastolic blood pressure), pneumonia-associated confusion, blood gas analysis (pH, partial pressure of oxygen (PaO2), partial pressure of carbon dioxide (PaCO2), fraction of inspired oxygen (FiO2)), chest radiograph (pleural effusion), and laboratory parameters (hematocrit, leucocyte count, band, serum creatinine, blood urea nitrogen, sodium, blood glucose). The patients were classified in risk classes according to the PSI. Blood samples were collected within the first 48 hours of hospitalization, after antibiotics initiation which doesn't affect the PSI score. The serum levels of 25-hydroxyvitamin D were determined by electrochemiluminescence binding assay in Roche Cobas 601 immunoassay analyzer and mean serum levels of 25-hydroxyvitamin D in each risk class were calculated. Vitamin D severe deficiency was defined as 25(OH)D levels <10 ng/ml, moderate deficiency as 25(OH)D levels between 10-20 ng/ml, and insufficiency as 25(OH)D levels between 20-29 ng/ml [19]. For statistical analysis, the statistical program SPSS for Windows version 17.0 (Statistical Package for the Social Sciences, Chicago, IL) was used. Continuous variables were tested for normality of distribution by the Kolmogorov-Smirnov test. For normally distributed values, descriptive results are presented as mean (standard deviation). One-way analysis of variance (ANOVA) test was used for the normal distribution variables. All p-values were two-sided and 5% was chosen as the level of statistical significance.

## Results

**Figure 1:**
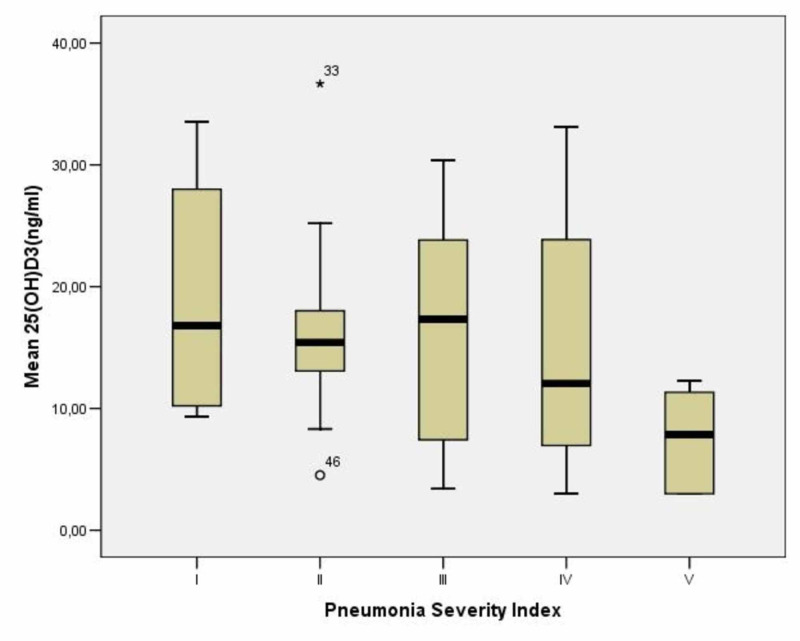
Correlation between mean 25(OH)D levels and PSI classes 33: Extreme Values; 46: Outlier PSI: pneumonia severity index

**Figure 2:**
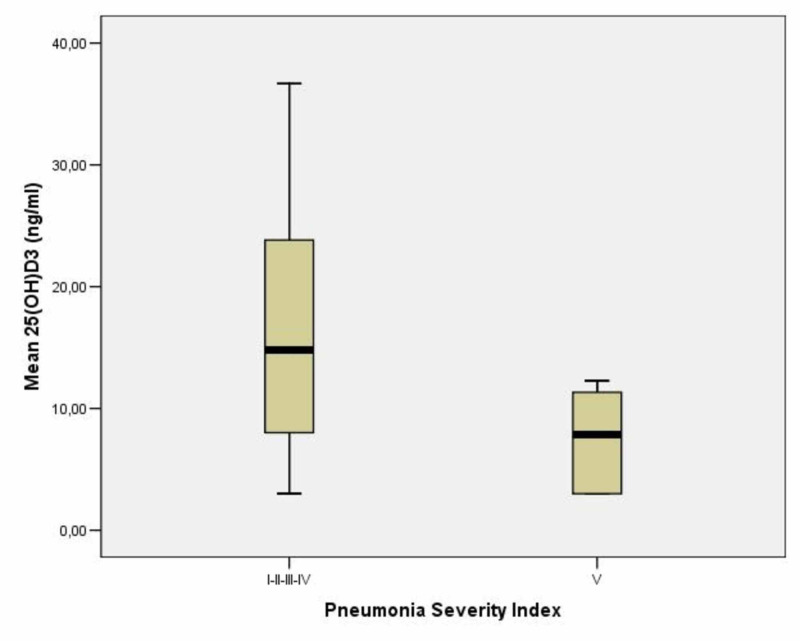
Mean levels of 25(OH)D in I, II, III, IV classes as compared to mean levels of 25(OH)D in class V Although for both PSI risk groups IV and V patients, the management is the admission of the patient and treatment with antibiotics and monitoring, class V is associated with the greatest mortality risk compared to classes I, II, III, and V. * p<0.05 PSI: pneumonia severity index

A total of 46 patients, 28 males and 18 females, with a mean age of 71.5±17.57 years and with community-acquired pneumonia, hospitalized from March 2019 to July 2019, were included. Verbal consent was obtained from all the patients. Sixteen patients (35%) had severe deficiency with 25(OH)D levels <10 ng/ml, 17 patients (37%) had moderate deficiency with 25(OH)D levels between 10 and 20 ng/ml and 13 patients (28%) had insufficiency with 25(OH)D levels between 20 and 29 ng/ml (Table 1). According to the PSI, four (8.7%) patients with mean age 53.75±15.43 years were classified as risk class I, 10 (21.7%) patients with mean age 54.7±14.82 years as class II, 10 (21.7%) patients with mean age 68.41±3.96 years as class III, 17 (37%) patients with mean age 84.82±9.73 years as class IV, and 5 (10.9%) patients with mean age 80.2±9.41 years as class V (Table 2). The mean levels of vitamin D were 19.11±11.24 ng/ml in class I, 16.81±8.94 ng/ml in class II, 16.65±9.18 ng/ml in class III, 14.76±10.22 ng/ml in class IV, and 7.49±4.41 ng/ml in class V (Table 2). There was a positive correlation between low levels of 25(OH)D and pneumonia severity (Figure 1) and statistically significant difference between mean levels of 25(OH)D in class V (7.49±4.41 ng/ml) compared to overall mean levels in class I, II, III and IV (16.15±9.49 ng/ml) with p<0.05 (Figure 2).

**Table 1:** Classification into severe deficiency, moderate deficiency, and insufficiency

Test 25(OH)D3	<10	10-20	>20
(ng/mL)	n=16	n=17	n=13
Anthropometric characteristics			
Age	74.75(20.84)	68.35(17.76)	71.61(17.57)

**Table 2:** Characteristics of study population and comparison of mean levels of 25(OH)D between PSI classes PSI: pneumonia severity index

PSI	I	II	III	IV	V
	n=4	n=10	n=10	n=17	n=5
Anthropometric characteristics					
Gender (male/female)	4/0	3/7	7/3	11/6	3/2
Age	53.75(15.43)	54.7(14.82)	68.4(13.96)	84.82(9.73)	80.2(9.41)
Test					
25(OH)D3	19.11(11.24)	16.81(8.94)	16.65(9.18)	14.76(10.22)	7.49(4.41)
	PSI I	Versus II	Versus III	Versus IV	Versus V
p		0.994	0.992	0.919	0.362
	PSI II	Versus I	Versus III	Versus IV	Versus V
p		0.994	1.000	0.982	0.380
	PSI III	Versus I	Versus II	Versus IV	Versus V
p		0.992	1.000	0.986	0.397
	PSI IV	Versus I	Versus III	Versus III	Versus V
p		0.919	0.982	0.986	0.554
	PSI V	Versus I	Versus II	Versus III	Versus IV
p		0.362	0.380	0.397	0.554

## Discussion

Pneumonia severity index (PSI) is used to classify the severity of pneumonia and to decide if patients with pneumonia can be treated as outpatients or need hospitalization. A risk class I or II patient with pneumonia can be treated at home with oral antibiotics (mortality risk of 0.1% and 0.6% within 30 days, respectively). A risk class III patient, after assessment of factors, including the circumstances and social climate conditions within the patient’s family and follow-up, may either be treated at home with oral antibiotics or be admitted for a short-term hospital stay (mortality risk of 2.8% within 30 days), while patients with risk class IV-V pneumonia should be hospitalized for treatment, as risk classes IV and V are associated with a mortality risk of 8.2% and 29.2% within 30 days, respectively [18].

A significant part of the antimicrobial activity of vitamin D is through the production of peptides that have antimicrobial activity and activity against endotoxins. Vitamin D triggers the expression of effective antimicrobial peptides, such as cathelicidin and β defensin, which occur in neutrophils, monocytes, natural killer (NK) cells, and epithelial cells covering the respiratory tract [20]. Immune cells such as macrophages, lymphocytes, and monocytes have vitamin D receptors (VDRs) that, with 25(OH)D stimulation, increase the expression of these peptides [21].

Cathelicidin is potent against gram-positive and gram-negative microorganisms, fungi, and mycobacteria at an assortment of pathogen entrance sites, including the skin and the respiratory and gastrointestinal mucosa [22]. Patients with 25(OH)D levels less than 20 ng/mL might be not able to completely express cathelicidin [23], which could be related to increased susceptibility to infections such as pneumonia and central line infections [24]. Another antimicrobial peptide, human beta-defensin-2 (HBD)- 2, may have exceptional utility in multidrug-resistant microorganisms from in vitro investigations [25]. Human beta-defensin-3 (HBD)- 3 is an antimicrobial peptide that has an extended range of antimicrobial action against gram-positive/negative microorganisms and fungi [26].

We found a positive correlation between low serum levels of 25-hydroxyvitamin D and the severity of community-acquired pneumonia in hospitalized patients assessed by PSI. The lowest levels of 25-hydroxyvitamin D were observed in class V, with a mean value of 7.49 mg/dl in class V as compared to an overall mean value of 16.14 mg/dl in classes I, II, II and IV (p<0.05). As, in our study, less than 30% of the patients had a specific pathogen isolated, we cannot report if there is any particular pathogen susceptibility in patients with lower vitamin D levels and if that correlates with the PSI score.

As mentioned above, the correlation between the levels of vitamin D and pneumonia severity assessed by the CURB-65 index has also been evaluated [16-17]. CURB-65, also known as the CURB criteria, is a clinical prediction rule that has been validated for predicting mortality in community-acquired pneumonia [27].

Brance et al. reported in their study that hospitalized adults with CAP have a high percentage of severe deficiency of 25(OH)D levels, which could lead to higher CAP severity [16]. Pletz et al. found a significant inverse correlation between the serum concentration of 1,25-OH2 vitamin D and the CURB score [17]. Talebi et al., in their prospective cohort study, examined 180 CAP patients admitted to a teaching hospital in Tehran, Iran, during 2016-2017 and found that the vitamin D status was lower in the more severe pneumonia cases than in the less (p=0.036) as assessed by the CURB-65 index. Vitamin D deficiency was more prevalent in patients who died than in those who lived while vitamin D concentration was negatively correlated with hospital stay duration and significantly greater in patients who survived than in those who did not (p<0.001) [28]. According to a study by Mamani et al., a low level of 25(OH)D was associated with a higher incidence of CAP and more severe disease assessed by the CURB-65 index [19].

The study has potential limitations. The patient's sample size is small due to these exclusions: patients <18 years old, severely immunocompromised patients, patients with tuberculosis, patients with malabsorption disorders, nursing home residents, patients with a history of malignancy, chronic renal or liver disease, patients with congestive health failure or cerebrovascular disease and patients receiving vitamin D as a supplement. Patients receiving vitamin D as a supplement were not enrolled because we mainly wanted to investigate if vitamin D levels are associated with mortality risk, without any exogenous administration of vitamin D. In this study, 73.9% of the patients included were > 60 years old. Although patients with diseases that can affect the PSI score were excluded, the majority of the patients were older, and old age has a great impact on PSI score. Older people have lower levels of vitamin D for several reasons such as poor skin integrity, reduced time spent outdoors, decreased intake of vitamin D, reduced renal function, and medications [29]. However, the important role of vitamin D deficiency in the development and severity of community-acquired pneumonia in the elderly has been reported in numerous studies. Lu et al. investigated the correlation between the levels of 25-hydroxyvitamin D and community-acquired pneumonia in elderly patients and concluded that the older patients with community-acquired pneumonia had a severe vitamin D deficiency, indicating that low levels of vitamin D might have an important role in the occurrence of community-acquired pneumonia [30].

## Conclusions

According to our results, there was a positive association between low levels of 25-hydroxyvitamin D and community-acquired pneumonia severity assessed by PSI. Aging is associated with a gradual decline in many aspects of the immune response, and waning immunity is thought to be an important risk factor for pneumonia in the elderly in combination with malnutrition and comorbidities. Although the treatment of pneumonia is antibiotics administration, the determination of 25-hydroxyvitamin-D status, mostly in patients >60 years old, during routine visits to primary care physicians, may prevent severe community-acquired pneumonia.
